# International Metric System of Weights and Measures

**Published:** 1876-11

**Authors:** 


### EXCERPTA.

---

### INTERNATIONAL METRIC SYSTEM OF WEIGHTS AND MEASURES.

In a memorial of "The International Association for obtaining a uniform system of Measures, Weights, and Coins," presented to Hon. Benjamin Disraeli, Chancellor of Her Majesty's Exchequor, in March, 1859, it is estimated that the adoption of the Metric System would save in the management of the London & Northwestern R. R. £10,000 ($50,000) a year; and in the various departments of Her Majesty's Government not less than £500,000 ($2,500,000) every year. When we consider that this computation was made by competent authorities, and remember that the amount named would be saved *every year* by a *single* railroad, it is evident that the total saving made by the adoption of the Metric weights and measures would be almost incredibly large. The same proportionate saving would be effected in every market, store, factory, counting-room, in short everywhere, for in business life nearly every sentence spoken or written contains some expression of quantity.

There has been recently incorporated in Boston an association of teachers and others interested in the introduction of the Metric weights and measures, under the name of the American Metric Bureau. Art. 2 of the constitution reads as follows:

"The object of this Bureau shall be to disseminate information concerning the Metric System; to urge its adoption; and to bring about actual introductions wherever practicable. To this end it will secure the delivery of addresses; publish articles; circulate books, pamphlets, and charts; distribute scales and measures; introduce the practical teaching of the system in schools; and in all proper ways, as far as the means at its disposal will allow, the Bureau will urge the matter upon the attention of the American people till they shall join the rest of the world in the exclusive use of the International Decimal Weights and Measures."

This Bureau already includes among its members many of our prominent educators, and its numbers are rapidly increasing. An office has been opened at 13 Tremont place, Boston, and as soon as suitable arrangements can be made, branch offices are to be opened in New York, Philadelphia, Chicago, and other central localities, where all persons interested are invited to call or to write freely in regard to any matter pertaining to the work of the Bureau.—*Met. Bureau.*

No country more than America feels the necessity of a uniform system of weights and measures. Her commercial and medical intercourse with three important European countries absolutely demands uniformity in this respect. In the adoption of a system that has been suggested, it will be seen that a notable deference to the computation of Federal money has been observed, in the decimal feature of the plan adopted. This and the metric system of France form the basis of the plan proposed by the International Convention, and there are abundant reasons for its speedy adoption by the American people. The following table of the Uniform System adopted, taken from Attfield's Chemistry, shows the conformity to our simple and easy plan:

### Units.

*Length.*—The *Unit of Length* is the Metre, derived from the measurement of the Quadrant of a Meridian of the Earth. (Practically it is the length of certain carefully preserved bars of metal, from which copies have been taken.)

*Surface.*—The *Unity of Surface* is the Are, which is the Square of Ten Metres.

*Capacity.*—The *Unity of Capacity* is the Litre, which is the Cube of a Tenth Part of a Metre.

*Weight.*—The *Unit of Weight* is the Gram, which is the Weight of that Quantity of distilled water, at its maximum density (40°C.), which fills a Cube of the One-hundredth part of the Metre.

### Table.

Note.—Multiples are denoted by the Greek words "Deka," Ten; "Hecto," Hundred; "Kilo," Thousand. Subdivisions by the Latin words "Deci," one-tenth; "Centi," One-hundredth; "Milli," one-thousandth.

| Quantities. | Length. | Surface. | Capacity. | Weight. |
|---|---|---|---|---|
| 1000 | Kilo-metre | . . . . | Kilo-litre | Kilo-gram |
| 100 | Hecto-metre | Hectare | Hecto-litre | Hecto-gram |
| 10 | Deka-metre | . . . . | Deka-litre | |
| 1 (Units) | METRE | ARE | LITRE | GRAM |
| .1 | Deci-metre | . . . . | Deci-litre | Deci-gram |
| .01 | Centi-metre | Centi-are | Centi-litre | Centi-gram |
| .001 | Milli-metre | . . . . | Milli-litre | Milli-gram |

When the metric method is exclusively adopted, these Units and Table, comprising the entire System of Weights and Measures, represent all that will be essential to be learned in lieu of the numerous and complicated tables hitherto in use. Adopting the style of elementary books on arithmetic, the tables may be expanded in the following manner.

| | | |
|---|---|---|
| 10 Milligrams | make | 1 centigram. |
| 10 Centigrams | " | 1 decigram. |
| 10 Decigrams | " | 1 gram. |
| 10 Grams | " | 1 dekagram. |
| 10 Dekagrams | " | 1 hectogram. |
| 10 Hectograms | " | 1 kilogram. |
| 10 Millilitres | " | 1 centilitre. |
| | etc. | |
| 10 Millimetres | make | 1 centimetre, |
| | etc. | |

The following approximate equivalents of metrical units should be committed to memory:

| | |
|---|---|
| 1 Metre | = 3 feet 3 inches and 3 eighths. |
| 1 Are | = a square whose side is 11 yards. |
| 1 Litre | = 1¾ pint. |
| 1 Gramme | = 15½ grains. |

The Metric Ton of 1000 Kilo-grammes = 19 cwt. 2 qrs. 20 lbs. 10 ozs.
The Kilo-gramme = 2 lbs. 3¼ ozs. nearly.
The Hect-are = 2½ acres nearly.

*Decimal Coinage.*—In most countries where the metric system of weights and measures is employed, a decimal division of coins is also adopted. This course, conjoined with the ordinary decimal method of enumerating, which fortunately is in universal use, renders calculations of all kinds most simple —easy to an extent which cannot be conceived in countries like England, where the operations of weighing, measuring, paying, and counting have only the most absurdly intricate relations to each other."

The following tables show that the international uniform system of weights and measures is based on the decimal plan, exactly that practiced in the computation of Federal money:

FEDERAL MONEY.

| | | |
|---|---|---|
| 10 Mills | make | 1 Cent. |
| 10 Cents | " | 1 Dime. |
| 10 Dimes | " | 1 Dollar. |
| 10 Dollars | " | 1 Eagle. |

METRIC MEASURES OF SURFACE.

| | | |
|---|---|---|
| 10 Millimeters | make | 1 Centimeter. |
| 10 Centimeters | " | 1 Decimeter. |
| 10 Decimeters | " | 1 Meter. |
| 10 Meters | " | 1 Dekameter. |
| 10 Dekameters | " | 1 Hectometer. |
| 10 Hectometers | " | 1 Kilometer. |

METRIC MEASURES OF CAPACITY.

| | | |
|---|---|---|
| 10 Milliliters | make | 1 Centiliter. |
| 10 Centiliters | " | 1 Deciliter. |
| 10 Deciliters | " | 1 Liter. |
| 10 Liters | " | 1 Dekaliter. |
| 10 Dekaliters | " | 1 Hectoliter. |
| 10 Hectoliters | " | 1 Kiloliter. |

METRIC WEIGHTS.

| | | |
|---|---|---|
| 10 Milligrams | make | 1 Centigram. |
| 10 Centigrams | " | 1 Decigram. |
| 10 Decigrams | " | 1 Gram. |
| 10 Grams | " | 1 Dekagram. |
| 10 Dekagrams | " | 1 Hectogram. |
| 10 Hectograms | " | 1 Kilogram. |

